# Fear conditioning in invertebrates

**DOI:** 10.3389/fnbeh.2022.1008818

**Published:** 2022-11-10

**Authors:** Amy K. Pribadi, Sreekanth H. Chalasani

**Affiliations:** ^1^Biological Sciences Graduate Program, University of California, San Diego, La Jolla, San Diego, CA, United States; ^2^Molecular Neurobiology Laboratory, The Salk Institute for Biological Studies, La Jolla, CA, United States

**Keywords:** learning, memory, invertebrates, fear conditioning, *C. elegans*, *A. californica*, *D. melanogaster*, predator-prey

## Abstract

Learning to identify and predict threats is a basic skill that allows animals to avoid harm. Studies in invertebrates like *Aplysia californica, Drosophila melanogaster*, and *Caenorhabditis elegans* have revealed that the basic mechanisms of learning and memory are conserved. We will summarize these studies and highlight the common pathways and mechanisms in invertebrate fear-associated behavioral changes. Fear conditioning studies utilizing electric shock in *Aplysia* and *Drosophila* have demonstrated that serotonin or dopamine are typically involved in relaying aversive stimuli, leading to changes in intracellular calcium levels and increased presynaptic neurotransmitter release and short-term changes in behavior. Long-term changes in behavior typically require multiple, spaced trials, and involve changes in gene expression. *C. elegans* studies have demonstrated these basic aversive learning principles as well; however, fear conditioning has yet to be explicitly demonstrated in this model due to stimulus choice. Because predator–prey relationships can be used to study learned fear in a naturalistic context, this review also summarizes what is known about predator-induced behaviors in these three organisms, and their potential applications for future investigations into fear conditioning.

## Introduction

Animals are challenged with threats throughout their entire life. Such threats may cause pain, which can be accompanied by actual damage to the animal’s body. Therefore, learning to avoid cues that predict threats and producing an appropriate behavioral response after encountering a noxious stimulus can help an animal avoid future harm. Given a changing environment, a nervous system should also be able to identify new threats as they arise and store these memories. Threats shape the evolution of behavioral programs and their underlying neural circuits and molecular mechanisms. In this review, we will discuss the study of fear conditioning in a laboratory with a focus on what has been accomplished in the invertebrates *Aplysia californica* and *Drosophila melanogaster*. Another invertebrate model organism, *Caenorhabditis elegans*, has not been used to study fear conditioning but we will discuss its use in similar conditioning schemes and its potential to use in fear conditioning research.

The term “fear” has many definitions and different facets of it are emphasized depending on the field of study. Fear is generally thought of as an emotional state whose outward expression can change depending on the species being studied. We will use a functional definition of fear similar to the one outlined in (Adolphs, 2013) which allows it to be applied across model organisms without needing to adhere to specific psychological correlates. According to this functional definition, fear as an internal state is caused by stimuli that can predict threat, and fear-elicited behaviors should allow the animal to mitigate that threat. The animal experiencing fear may already have an innate response to certain stimuli that does not require training—for example, rats avoid cat odor even without experiencing cat attacks (Blanchard et al., 1990; Dielenberg et al., 2001)—but experience can also teach the animal that a previously neutral stimulus is predictive of threat. In this review, we will focus on the stimuli used in the training protocol in the context of the animal’s natural habitat as well as whether the elicited behavior makes adaptive sense rather than the internal representation of fear itself.

Learning can be either non-associative or associative (Carew and Sahley, 1986). Non-associative learning involves changing behavior in response to repeated presentations of a single stimulus. Non-associative forms of learning include habituation, where the animal’s response to repeated presentations of the same stimulus diminishes over time, and sensitization, where the animal’s response to a stimulus increases after repeated exposures. In contrast, associative learning involves learning a new relationship between two stimuli (Carew and Sahley, 1986). Associative learning typically allows the animal to assign new values to stimuli so that they can learn about new threats or contexts.

The systematic study of associative learning historically utilizes classical conditioning (Rescorla, 1967, 1968), which involves using a stimulus that evokes a “hard-wired” behavioral response, such as freezing in response to pain. This is called the unconditioned stimulus (US) because no prior conditioning is required to elicit the behavioral response. The experimenter then tests to see if the animal can associate an unrelated stimulus with the US through temporal pairing of the two stimuli. The new stimulus is called the conditioned stimulus (CS) because the animal requires conditioning to respond to it. After successful training, when presented with the CS alone the animal will behave as if it is receiving the US. The interpretation of these results is that, through training, the animal learns that the CS predicts the US. The probability of the CS predicting the US is directly correlated with learning ability (Rescorla, 1967, 1968; rev. in Carew and Sahley, 1986). Allowing time between CS-US training trials (spaced training) produces long-lasting memory, while grouping all CS-US trials together (massed training) tends to only result in immediate-term memory (e.g., Yin et al., 1995; Sutton et al., 2002; Amano and Maruyama, 2011). Fear conditioning is a type of associative learning where the unconditioned stimulus is one that can elicit fear. Mild electric shock that causes pain to the animal is often used in these studies. The unconditioned response to the fear-inducing cue is usually a defensive reflex, like jumping or freezing.

While classical conditioning can test the extent to which animals are capable of pairing two arbitrary stimuli, learning occurs in the context of the natural environment in which the animal has evolved. This means that different species should be more equipped to learn some tasks better than others depending on what cues are chosen. For example, rats are more likely to associate pain from shock with audiovisual cues, not gustatory cues. Conversely, they are more likely to associate nausea-inducing X-rays with taste, not audiovisual cues. This makes sense intuitively; audiovisual cues often precede attacking predators that induce pain, while ingested cues can precede eating toxins that cause nausea (Garcia and Koelling, 1966). The sensory modality of the cues thus affects the ability for the organism to learn from them, as it depends on the organisms’ innate abilities as well as its natural environment.

Studying animals with simple neuroanatomy and robust behaviors allows these biological processes to be analyzed at the level of individual neurons, circuits and molecular pathways. The usefulness of invertebrates in the laboratory has led to illuminating discoveries in the field. With this in mind, we will review some major findings in fear conditioning using invertebrate model organisms. We will first describe major findings obtained using studies in the sea slug *A. californica*, the fruit fly *D. melanogaster*, and in the nematode *C. elegans.* We will also summarize the similarities and differences between all three organisms and discuss how current models of learning in invertebrates fit into the broader study of fear conditioning. We will conclude this review with musings on how fear conditioning fits into more naturalistic setups, especially using natural predators as a threat.

## Aplysia californica

Early experiments in invertebrate learning were done in the sea slug *A. californica*. This animal was selected due to its relatively few neurons (∼20,000) and the fact that its neurons were easy to record from owing to their large size (Kandel, 2001). This model organism, specifically the gill-withdrawal circuit, helped to identify mechanisms of non-associative learning and memory. Repeated stimulation of the gill-withdrawal reflex diminishes its response (habituation) but a single shock to the head brings back the response (dishabituation) without activating sensory neurons within the same circuit (Castellucci and Kandel, 1976). The gill and siphon withdrawal circuit also demonstrates sensitization, as a single noxious stimulus to the circuit enhances the withdrawal reflex for several minutes (Kandel and Schwartz, 1982; Yovell et al., 1987). Further studies showed that this type of short-term learning arises from a presynaptic change in calcium current rather than any structural change in the number of synapses within the circuit (Klein and Kandel, 1980). Through these experiments, it became clear that past experience of a neural circuit can alter the strength of signaling at the synapse through rapid intrinsic mechanisms, allowing for short-term plasticity that does not require physical re-wiring of the existing circuit.

*Aplysia* also served as a useful model to determine the molecular basis of these intrinsic mechanisms. Studies found that serotonergic signaling increased cAMP levels (Cedar and Schwartz, 1972), which in turn activated PKA and altered presynaptic neurotransmitter release by increasing calcium influx (Castellucci and Kandel, 1976; Klein and Kandel, 1980; Kandel, 2001). These mechanisms can account for short-term behavioral effects without the need for new proteins to be synthesized. In contrast, memory that lasts beyond a day requires transcription and translation during the training period (Goelet et al., 1986; Castellucci et al., 1989). Furthermore, cell culture studies showed that serotonin induces phosphorylation of the transcription factor CREB-1, which acts in the nucleus to induce transcription of selected genes that enforce long-term memory, providing a molecular link between short-term and long-term sensitization (Kandel, 2001).

**FIGURE 1:**
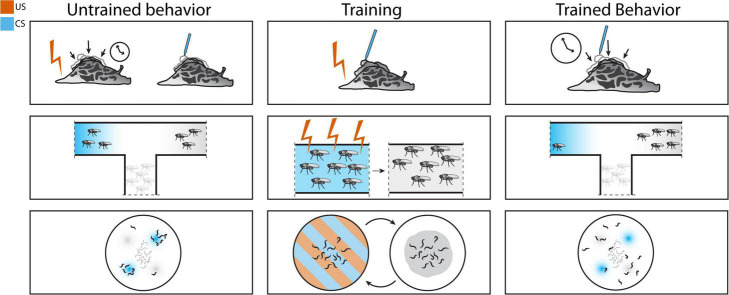
Associative conditioning schemes in *Aplysia* **(top)**, *Drosophila* **(middle)**, and *Caenorhabditis elegans* **(bottom)**. Classical conditioning in *Aplysia*: training is performed by pairing electric shock with siphon touch. After training, siphon touch elicits the longer whole-body withdrawal response normally seen only with electric shock. Olfactory fear conditioning in *Drosophila melanogaster*: pairing electric shock with odor can alter olfactory preference. Aversive olfactory conditioning in *C. elegans*: pairing acid with odor results in avoidance of the paired odor.

In addition, *Aplysia* can form associative memories through classical conditioning. Using the siphon- and gill-withdrawal reflex as a test circuit, a light touch to the siphon (CS) produces a weak siphon withdrawal while a strong shock to the tail (US) produces a strong, longer-lasting withdrawal. The withdrawal response to light siphon touch is three times longer in animals trained with paired CS-US. Increasing the number of trials produces longer withdrawal responses, and the learned association is extinguished within ten trials of CS without US. This is a slight variation on classical conditioning as the elicited response is not a new US-evoked behavior, but rather a stronger version of the CS-evoked behavior (Carew et al., 1981). This training scheme is summarized in Figure 1. *Aplysia* can also form associative memories with odor. *Aplysia* are able to associate shrimp odor (CS) with head shock (US) proficiently after five trials (Walters et al., 1979). Studies using electrophysiological assays showed that the same activity-dependent presynaptic facilitation mechanisms identified in non-associative learning paradigms also play a role in associative learning (Hawkins et al., 1983). During short-term sensitization, serotonin acts through PKA to facilitate presynaptic neurotransmitter release.

Other researchers have demonstrated a role for postsynaptic mechanisms in associative learning in *Aplysia* for response specificity (Glanzman, 1995, 2008). The advantage of *Aplysia* is the ease of recording and direct electrical or chemical manipulations in their large neurons, but experiments on dissected preparations are not easily comparable with behavioral studies with intact, behaving animals (Glanzman, 1995). Model organisms that are more tractable to genetic manipulation were instrumental in making this connection.

## Drosophila melanogaster

The high fecundity and short reproductive cycle of the fruit fly *D. melanogaster* make it an indispensable tool in identifying the genetic components of learning. Forward genetic screens identified many genes involved in different aspects of learning. Behavioral assays can also be conducted on populations of flies, allowing for more powerful studies. The development of tools for conditional gene expression unlocked the ability to explore the temporal and spatial specificity of learning and memory. The yeast-derived GAL4-UAS system and its variants enabled spatial control of transgene expression (Brand and Perrimon, 1993; Duffy, 2002). Genetic screens have yielded libraries of mutants, including temperature sensitive mutants. An especially useful temperature mutation in *shibire*, a Dynamin ortholog, enabled circuit tracing with temporal control. Growing flies at a permissive temperature allows normal function of the gene. Shifting to the restrictive temperature blocks neurotransmission in neurons expressing *shibire^ts^* (van der Bliek and Meyerowrtz, 1991). The combination of selective gene expression and temperature sensitive mutations grants control of neural activity with both spatial and temporal specificity, and allowed researchers to study how different regions of the *Drosophila* brain contribute to learning and memory.

*Drosophila* are capable of associative learning by pairing specific odors with shock. Using the odorants 3-octanol or 4-methylcyclohexanol as CS and electric shock as US, researchers found that flies specifically avoid entering tubes with the shock-associated odor. Memory of this training persists for at least an hour, and four spaced training events separated by two hours is sufficient to induce memory for at least a day (Quinn et al., 1974). This protocol was later modified to use a T-maze as a learning test after a training cycle of 60s odor exposure with or without inescapable shock followed by 30s of rest, and exposure to the second odor with or without shock. Learning in the T-maze is determined by comparing the number of flies in the two collection tubes with either shock-paired odor or unpaired odor. This method of conditioning has a higher success rate, with maximal training achieved after a single training cycle (Tully and Quinn, 1985). This protocol (Figure 1) is still widely used in memory studies (Keene and Waddell, 2007).

A complete dissection of the circuits involved in fear conditioning to odorants requires identifying the pathways coding both odor and electric shock, as well as identifying the site of integration. The pathway for odor sensing can be summarized as follows: olfactory sensory neurons (OSNs) from the antennae and maxillary palps project to glomeruli within the antennal lobe, and projection neurons (PNs) from the antennal lobe form synapses with Kenyon cells in the mushroom body (MB) and terminate in the lateral horn. Within the antennal lobe, there are additional local excitatory and inhibitory interneurons that connect to the OSNs and span multiple glomeruli. The specifics of the olfactory circuit, particularly the anatomy of the MB, has been reviewed in-depth multiple times (Margulies et al., 2005; Keene and Waddell, 2007; Scheffer et al., 2020). Ablation of the MB results in memory defects but spares naïve avoidance of odors (de Belle and Heisenberg, 1994), so the MB appears to be an important site of integration downstream of odor sensation. The circuit for sensing electric shock is not known, but aminergic signaling is likely involved, consistent with results from *Aplysia* where shock triggers serotonin release. One study expressed the fluorescent calcium indicator cameleon in dopaminergic neurons and found that dopaminergic projections into the MB are activated by electric shock, and in trained flies these responses are prolonged for odor paired with shock (Riemensperger et al., 2005). Another study utilizing both *shibire^ts^* and the GAL4/UAS system showed that blocking neurotransmission from dopaminergic neurons blocks aversive, but not appetitive, olfactory learning (Schwaerzel et al., 2003). *Drosophila* can also demonstrate reversal learning, where flies can switch learned odor avoidance. Flies are first trained with two different odors. one presented with shock and one without. Then, in a reversal training period, the pairing of specific odor and shock was switched. After the first training period, flies avoid the shock-associated odor. After both training periods, flies show reduced avoidance of the first shock-associated odor and aversion of the second shock-associated odor. Reward-associated dopaminergic neurons are activated when shock is omitted during reversal training, and activation of punishment-encoding dopaminergic neurons is reduced (McCurdy et al., 2021). These studies suggest that dopamine, rather than serotonin, is the reinforcing signal for aversive learning during olfactory conditioning with electric shock.

Furthermore, unbiased genetic screens in *Drosophila* identified similar components to learning and memory as those in *Aplysia* using the aversive olfactory condition protocol explained above. The cAMP/PKA pathway was identified in screens for learning and memory deficient flies (Dudai et al., 1976; Byers et al., 1981; Livingstone et al., 1984; Nighorn et al., 1991). In particular, the gene *rutabaga* encodes a type I Ca^2+^/calmodulin-stimulated adenylyl cyclase that is theorized to be an important coincidence detector through detecting both Ca^2+^ increase and G protein signaling following monoamine binding (Connolly et al., 1996; Keene and Waddell, 2007). Expression of *rut* cDNA in the MBs can rescue the memory deficient loss of function mutant (Zars et al., 2000; McGuire et al., 2003).

## Caenorhabditis elegans

An organism with even fewer neurons, the hermaphroditic nematode *C. elegans* is yet another useful model for studying neural mechanisms. *C. elegans* in nature are found in rotting organic material, such as fruits and stems, where they feed on the diverse microbes they encounter (Schulenburg and Félix, 2017). In the laboratory, *C. elegans* is grown in axenic culture with *Escherichia coli* OP50 as food. Like with fruit flies, *C. elegans* behavioral studies are usually conducted with populations of whole, behaving animals rather than dissected preparations. With 302 neurons and a mapped connectome (White et al., 1986; Cook et al., 2019; Brittin et al., 2021; Witvliet et al., 2021), *C. elegans* is an excellent model to study neural circuits on a single-cell basis. It also is a convenient genetic model due to its ability to package injected DNA into extrachromosomal arrays (Mello et al., 1991; Mello and Fire, 1995), generating transgenic lines in a short period of time. The history of *C. elegans*’ use as a genetic model also means that there exist libraries of mutants and lists of cell-specific promoters ready for use in experiments. While its neurons may be too small to easily patch, genetically encoded fluorescent calcium indicators can be easily imaged through its transparent body to study neural activity.

While the studies in *Drosophila* and *Aplysia* have explored fear conditioning by using electric shock as US, most aversive conditioning schemes in *C. elegans* involve avoiding starvation which is different than avoiding cues that predict pain or bodily harm. However, starvation-associated protocols have been used to model aversive associative learning, setting the foundation for probing fear learning in *C. elegans*. Briefly, odors such as 2-butanone and benzaldehyde, which are sensed by the AWC neurons, are normally attractive (Bargmann et al., 1993). If a population of *C. elegans* experiences one of these attractive odors in the absence of food, they will no longer find that odor attractive (Colbert and Bargmann, 1995; Nuttley et al., 2002). The extent of behavioral change increases with increased training time and plateaus at 90 min (Colbert and Bargmann, 1995). As in *Aplysia* and *Drosophila*, aminergic signaling has been shown to be involved during the training. Adding serotonin during odor conditioning mimics the effect of adding food and blocks aversive learning. Mutants in *cat-4* that lack both serotonergic and dopaminergic signaling have normal naïve approach and decreased attraction after training with starvation, and adding food does not disrupt their training (Torayama et al., 2007).

*Caenorhabditis elegans* show variable length of memory retention depending on the duration of training. *C. elegans* trained for 10 min exhibits a mild decrease in attraction, while those trained for 60-90 min ignore the odor for at least 150 min (Lee et al., 2010). This length of memory is dependent on the cGMP-dependent protein kinase, EGL-4 (LEtoile et al., 2002). GFP-tagged EGL-4 enters the nucleus in the AWC neurons immediately after odor conditioning, suggesting that its nuclear translocation initiates learning (Lee et al., 2010). Later research showed that EGL-4 phosphorylates proteins in the nucleus that promote the sustained change in behavior through RNA interference, providing a molecular link between early events during training and memory retention (Juang et al., 2013). The memory of food status and odor associative training can be pushed even longer through spaced training, allowing the study of the mechanisms of long-term memory. Enhanced attraction to butanone due to association with food normally lasts around two hours, but the length of this memory can be increased by training in spaced blocks of food-butanone exposure separated by periods of starvation. After seven such training blocks, memory as indicated by enhanced attraction remains for 16 h. Cycloheximide and actinomycin D treatment block this long-term memory but not immediate memory, indicating that transcription and translation are required for the formation of long-term memory. Better long-term memory is also associated with increased levels of phosphorylated CREB (Kauffman et al., 2010).

In a starvation-based assay, it can be argued that the absence of food rather than the presence of starvation induces aversive learning. The absence of a positive stimulus is not the same as an aversive stimulus. Therefore, while *C. elegans* may be able to learn to avoid cues predictive of unfavorable environments, this is not quite the same as the fear conditioning schemes mentioned in other organisms. Other noxious stimuli used in aversive training paradigms include acid (Amano and Maruyama, 2011). *C. elegans* will avoid acidic conditions of pH 4.0 or lower, regardless of growth condition (Sambongi et al., 2000), suggesting that this avoidance is innate rather than learned, as *C. elegans* are known to gravitate toward temperature (Hedgecock and Russell, 1975) and salt concentrations (Kunitomo et al., 2013) that match their growth condition. Investigators used HCl pH 4.0 as a CS and a normally attractive odorant, 1-propanol, as a US. Pairing the HCl and 1-propanol by soaking the *C. elegans* in a solution containing both lead to avoidance of 1-propanol in a chemotaxis assay (Figure 1), and maximum learning was achieved after five conditioning cycles. Temporal separation of the two cues (inter-stimulus interval, ISI) affected the degree of change in learned behavior; more time in between cue presentations resulted in less learned behavioral change. The duration of memory was also affected by the time between training cycles (inter-trial interval, ITI). While learning was demonstrated immediately after training with 0 min ITI, memory was retained for 3 h after a 10 min ITI. Adding translational or transcriptional inhibitors blocked both short-term and long-term learning in a spaced training protocol. These inhibitors did not block short-term learning in a massed training protocol, which normally does not induce long-term learning. Mutants in *crh-1* (CREB), *glr-1* (AMPA receptor), and *nmr-1* (NMDA receptor) all showed decreases in long-term learning. This study shows that *C. elegans* can associate a noxious stimulus with a cue and avoid that cue (Amano and Maruyama, 2011).

While acid can cause pain (Wemmie et al., 2013) and potential harm (Cong et al., 2020) to the *C. elegans* cuticle, can it be considered a fear-inducing stimulus? It is difficult to technically parse threat types especially when *C. elegans* avoidance behavior can look similar across many aversive stimuli. However, an acidic environment is fundamentally different from a physical attack represented by electric shock; while both may cause pain and/or harm, an acidic environment has a spatial restriction that does not necessitate learning predictive cues in the same way as physical attacks received in other animals. Therefore, while aversive conditioning studies done in *C. elegans* demonstrate that it can be used to model learning, fear conditioning in this organism has still not been demonstrated.

## Predators and fear conditioning

Naturalistic fear-related behaviors can be studied in the context of predator-prey relationships. Live cats (Blanchard et al., 1990) and cat odors (Dielenberg et al., 2001) have been used in rodent studies. As mentioned above, the choice of fear-inducing stimulus is an important step, which requires consideration of the model organism’s natural environment and the types of predators that they may encounter. Also, predator behavior influences which prey behavioral strategies maximize survival. For example, prey of nocturnal predators reduce their feeding as nighttime approaches with increased risk of predation (Clarke, 1983). While prey behavior affects survival in context of differing predation strategies, prey must also balance their other needs: foraging and reproduction. This cost-benefit calculation is likely a driving force in the evolution of prey behavior (Lima, 1998; Abrams, 2003; Lima and Bednekoff, 2015).

Investigators have already used predators to study fear learning in *Aplysia*. The spiny lobster *Panulirus interruptus* can attack and bite *Aplysia*. A single attack bout sensitizes the withdrawal reflex of both head and tail-mantle (Watkins et al., 2010). Multiple bouts lead to long-term sensitization that lasts for at least 24 h. Sensory neurons in *Aplysia* that has been attacked multiple times have a lower threshold to produce action potentials (Mason et al., 2014). The sensitization of these withdrawal reflexes could potentially make them less accessible to predation. Previous studies have shown that electric shock also results in long-term sensitization via increased sensory neuron excitability (Scholz and Byrne, 1987; Walters, 1987; Cleary et al., 1998), similar to the effect of lobster attack in these studies. Another study analyzed behavior following predator attack; attacks from spiny lobster or blue crab (*Callinectes sapidus*) suppressed feeding behavior temporarily but normal feeding behavior resumed after two hours (Wolfe et al., 2016). Another predator, a sea slug *Navanax inermis*, utilizes a different form of attack. Rather than biting, *Navanax* squeezes its prey. Investigators found that *Navanax* attacks to the head results in sensitization of the head, and to a lesser extent, a tail-mantle withdrawal. However, multiple spaced *Navanax* attacks failed to produce long-term sensitization in the head sensitized tail mantle withdrawal (Pepino et al., 2022). These studies show that predator attacks mimics some of the effects of electric shock in terms of sensitization, although the nuances of memory length and site of sensitization can be different.

*Drosophila* predators can include hunting insects like pantropical jumping spider *Plexippus pakulli* and the Texas unicorn mantis *Phyllovates chlorophaena*. When housed in a circular arena with *Drosophila*, both predators elicit avoidance in *Drosophila* when compared to non-predatory insects. *Drosophila* relies on visual cues to detect these predators—mutants with visual impairment no longer show predator avoidance. Additionally, avoidance can also be elicited using mock predators with similar visual characteristics to the insect predators; flies avoid a moving mock spider (de La Flor et al., 2017). *Drosophila* are also known to avoid looming stimuli—rapidly expanding visual stimuli that mimic approaching threats. Numerous studies have elucidated the neural circuits from vision to motor neurons that underlie escape responses elicited by looming stimuli (de Vries and Clandinin, 2012; Muijres et al., 2014; von Reyn et al., 2014; Ache et al., 2019; Morimoto et al., 2020). These studies demonstrate innate avoidance of visual traits that predict predator threat, but they do not yet explore if exposure to predators leads to learned behaviors. Moreover, a looming stimulus is different from the physical attack explored in *Aplysia* because the visual stimulus itself is not harmful; it may be interesting to combine the predator-associated visual stimulus with electric shock. A rat study took this approach by simulating an owl attack with both a visual looming stimulus and electric shock. This study found that a pairing of looming/shock with an auditory CS could induce escape behavior upon auditory tone presentation. However, rats trained with only CS and shock or CS and looming did not display escape behavior upon CS presentation. Interestingly, rats exposed to looming and shock but not the CS still displayed reduced foraging behavior upon the novel tone presentation, implying that non-associative learning after threat presentation can be a major factor in naturalistic environments (Zambetti et al., 2022).

Many types of fungi prey upon nematodes like *C. elegans*. Some fungi send out structures made of hyphae that can trap, paralyze, and digest the live nematodes. A 100-million-year-old fossilized sample of a nematode caught in a carnivorous fungi trap indicates that the relationship between nematodes and carnivorous fungi is ancient and widespread (Schmidt et al., 2007). Through this coevolution, fungi developed advantageous mechanisms including luring their nematode prey with volatile cues that mimic food (Hsueh et al., 2017) and sensing pheromones released by *C. elegans* to increase trap formation (Hsueh et al., 2013). *C. elegans* also has evolved mechanisms to escape death by fungi. Fungi like *Drechslerella doedycoides* send out constricting rings at the end of some hyphae that can catch *C. elegans* that crawl through them. The three cells that form the ring must inflate to successfully trap the nematode, but wild-type worms sense the ring and respond quickly enough to escape most of the time. The anterior touch response, which includes the worm both backing up and suppressing head movement, enables escape. Tyramine signaling is required to coordinate this behavior and tyramine-gated chloride channel mutants fail to escape carnivorous fungi as often as wild-type worms in a direct competition assay (Maguire et al., 2011). The study of *C. elegans’* behavioral responses to carnivorous fungi are an example of using *C. elegans* to identify genes important to a population’s survival in when faced with a natural predator.

Another predator of *C. elegans* is the nematode *Pristionchus pacificus. C. elegans* is known to be found in decaying organic matter such as stems or fruits (Schulenburg and Félix, 2017). A study surveying the environments where *Caenorhabditis* are commonly found also discovered *P. pacificus* in samples of rotting stems and fruits (Félix et al., 2018). *P. pacificus*, like *C. elegans*, is a bacterivorous hermaphrodite. Unlike *C. elegans, P. pacificus* is also a facultative predator of other nematodes. *P. pacificus*, as well as other species in the *Pristionchus* genus, have tooth-like structures that enable it to bite and consume their prey. *P. pacificus* show environmentally-influenced polymorphism in their tooth development. Depending on culture conditions, some develop into a narrow-mouthed stenostomatous (St) morph with a single tooth while others develop into a wide-mouthed eurystomatous (Eu) morph with both a dorsal and a ventral tooth, which facilitates predation of other nematodes (Serobyan et al., 2014). We have previously shown that *C. elegans* can sense sulfolipids secreted by *P. pacificus* and avoids them (Liu et al., 2018). *P. pacificus* could be an interesting predator to evoke fear responses because, unlike carnivorous fungi, they can inflict sub-lethal damage. *P. pacificus* is a relatively proficient killer of *C. elegans* larvae but they fail to kill *C. elegans* adults despite biting them at the same rate (Wilecki et al., 2015). The bite of a predator like *P. pacificus* could be used as an unconditioned stimulus. The study of various *Pristionchus* species has primarily been used to study the evolution of features such as mouth form development and neural circuit pattern/function. We suggest that the *Pristionchus*—*Caenorhabditis* interaction might also be leveraged to study learned fear in nematodes.

## Discussion

Research into learning and memory using these three invertebrate models has revealed shared patterns of memory acquisition and similar molecular players. Table 1 shows a comparison between experimental setups in fear conditioning or fear condition-like protocols across the three invertebrates discussed above.

**TABLE 1:** A comparison of fear conditioning or fear conditioning-like schemes in each organism.

Organism	Training stimuli	Behavioral test	Recall time
*Aplysia californica*	Siphon touch (CS) and electric shock to tail (US)	Extended siphon withdrawal time	With spaced training, over a day
*Drosophila melanogaster*	Odor (CS) and electric shock (US)	T-maze (avoidance of US-paired odor)	Over an hour; with spaced training over a day
*Caenorhabditis elegans*	Odor (CS) and acid (US)	Chemotaxis assay (avoidance of odor)	Up to 24 hours with spaced training

Each model organism has its unique benefits. For example, *Aplysia* researchers benefit from this model’s relatively large neurons, which can be directly recorded from and manipulated with drugs or neurotransmitters. Genetic manipulation in *Drosophila* research has illuminated the molecular pathways that contribute to the different phases of memory, and genetic tools have allowed mapping the activity of these genes to specific places within the relevant circuits. *C. elegans* have confirmed the genetic findings in *Drosophila* but so far have been underutilized in fear conditioning-like experiments. A potential technique that is relatively easy in *C. elegans* compared to other organism is imaging neuronal calcium activity during behavior, as its transparent body allows visualization of calcium indicators without requiring invasive surgeries or restricting movement (Nguyen et al., 2016). This technology is still relatively new and still being developed, both in terms of microscopy equipment and data interpretation particularly when simultaneously imaging all neurons. However, we believe this technique in *C. elegans* could be leveraged in the future to identify how training schemes modify the state of the entire nervous system.

**FIGURE 2:**
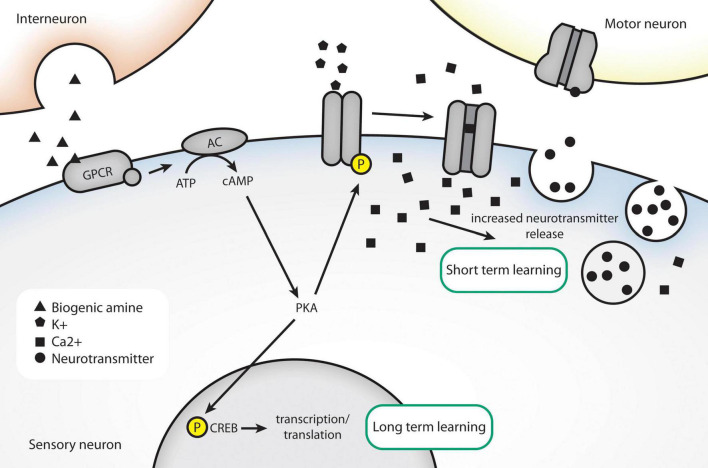
Conserved molecules in associative learning, as identified in invertebrate models. An unconditioned stimulus, like electric shock, causes release of dopamine/serotonin. The presence of a conditioned stimulus causes postsynaptic calcium levels to rise through closing of K+ channels. High intracellular calcium results in increased release of neurotransmitter. Long-term behavioral changes can be effected through altered transcription via nuclear translocation of CREB.

Each of these models has shown a similar ability to undergo aversive conditioning. The length of memory also depends on the training scheme; generally, spaced training induces long-term memory, while massed training does not. The underlying molecules that encode immediate-term and long-term learning are shared between all three. For example, mechanisms of immediate-term memory in all three organisms involve signaling via cAMP/PKA and are independent of new protein synthesis. In *Aplysia*, short-term sensitization of the gill-withdrawal circuit involves alteration of presynaptic current (Armitage and Siegelbaum, 1998). In *Drosophila*, short-term memory of olfactory conditioning is lacking in mutants in *rutabaga*, a type I Ca^2+^/calmodulin dependent adenylyl cyclase (Levin et al., 1992; Zars et al., 2000). In contrast, long-term learning appears to require transcription and translation as inhibitors like cycloheximide and actinomycin D inhibit long-term learning and the homologs for the transcription factor CREB are required specifically for long-term learning, or learning induced by massed training protocols (Bartsch et al., 1995; Esdin et al., 2010; Kauffman et al., 2010; Amano and Maruyama, 2011). A summary of the molecules involved in forming short-term and long-term memory are included in Figure 2. Induction of a CREB activator isoform in *Drosophila* could produce long-term memory after a single training bout (Yin et al., 1995).

Biogenic amines like serotonin and dopamine have been shown to play a crucial role in acquiring and storing information in all three invertebrate models. While serotonin levels can encode food status in nematodes (Nuttley et al., 2002) and modulates gill-withdrawal after aversive conditioning in *Aplysia* (Castellucci and Kandel, 1976), dopamine reinforces punishment in the fly model (Riemensperger et al., 2005). Multiple studies have shown roles for these biogenic amines in mammalian learning and memory as well (e.g., Rake, 1973; González-Burgos and Feria-Velasco, 2008; Meneses and Liy-Salmeron, 2012; Clos et al., 2018).

While electric shock has the benefit of control of stimulus delivery, which allows temporal control between CS and US delivery, it is not one that animals likely encounter in its natural habitat. Therefore, the interpretation of traditional CS/US fear conditioning experiments in the context of natural behavioral adaptations can be difficult. Exploration of naturally occurring threats to *Aplysia* shows that sublethal predator attacks can induce short- and long-term sensitization of multiple reflexes, and these results are similar to those results from electric shock though some aspects of memory duration and location of sensitization can differ (Watkins et al., 2010; Mason et al., 2014; Wolfe et al., 2016; Pepino et al., 2022). *Drosophila*’s avoidance of visual stimuli that mimic predator approach could also potentially be used to explore learning in *Drosophila*, especially if paired with electric shock. In *C. elegans*, mechanosensory circuits have been shown to undergo habituation, dishabituation, and sensitization (Rankin et al., 1990). The rate of habituation can be altered in a context-dependent way; when on food, dopamine slows down the rate of tap habituation (Sanyal et al., 2004). *C. elegans* use mechanosensation to detect and escape predatory fungi (Maguire et al., 2011). These discoveries in mechanosensory learning could potentially be applied to studies using predators such as predatory fungi or predatory nematodes.

Studies in invertebrates have shown similar mechanisms behind learning and memory. Associative learning in response to pain from electric shock in *Aplysia* and *Drosophila* or a damaging acidic environment in *C. elegans* have demonstrated fear conditioning or fear conditioning- like learning. While fear conditioning is generally associative, using predators and their effect on defensive behaviors could provide insight into additional mechanisms of non-associative learning.

## Author contributions

AP wrote an initial draft of the manuscript, which was edited by SC. Both authors contributed to the article and approved the submitted version.
